# Effects of Chewing Different Flavored Gums on Salivary Flow Rate and pH

**DOI:** 10.1155/2012/569327

**Published:** 2012-03-11

**Authors:** Maryam Karami Nogourani, Mohsen Janghorbani, Raha Kowsari Isfahan, Mozhgan Hosseini Beheshti

**Affiliations:** ^1^Department of Pediatric Dentistry, Faculty of Dentistry, Islamic Azad University, Khorasgan Branch, Isfahan, Iran; ^2^Department of Epidemiology and Biostatistics, School of Public Health, Isfahan University of Medical Sciences, 8144503500 Isfahan, Iran

## Abstract

Chewing gum increases salivary flow rate (SFR) and pH, but differences in preferences of gum flavor may influence SFR and pH. The aim of this paper was to assess the effect of five different flavors of sucrose-free chewing gum on the salivary flow rate and pH in healthy dental students in Isfahan, Iran. Fifteen (7 men and 8 women) healthy dental student volunteers collected unstimulated saliva and then chewed one of five flavored gums for 6 min. The whole saliva was collected and assessed for 6 consecutive days. After unstimulated saliva was collected, stimulated saliva was collected at interval of 0-1, 1–3, and 3–6 minutes after the start of different flavored chewing gums. The SFR and salivary pH were measured. The SFR increased in all five flavored gums at 1, 3, and 6 minutes after start of chewing gums (*P* < 0.001). The flow rate of all products reached peak in the 1st minute of stimulation, except spearmint-flavored gums which reached peak in the 6th minute. In the 1st minute, the strawberry-flavored gums showed the highest SFR. During 1–3 minutes, strawberry- and apple-flavored gums showed higher SFR, respectively. Only the spearmint- and cinnamon-flavored gum significantly increased salivary pH. Gum flavored can affect the SFR and pH and special flavors can be advised for different individuals according to their oral conditions.

## 1. Introduction

Saliva is important for oral and dental health, because increasing salivary flow rate (SFR) increases pH, promotes enamel remineralization and buffer capacity, and reduces caries [1]. Chewing sucrose-free gums is a convenient way to increase salivary flow and the oral health benefits of gum chewing are well known [2]. Gum chewing increases salivary flow through a combination of gustatory and mechanical stimulation [3]. It has been shown that on chewing flavored gum, the salivary flow rate increases initially but declines as the flavor is lost from the gum, and as the gum softens with chewing [4, 5]. Nowadays, many kinds of chewing gum, with different flavors, shapes, and commercial packages, are available and are selected according to personal taste. It has been shown that chewing gum taste is an important factor in individual's preference, and gum selection can influence on long-term compliance [6]. There is a dearth of information available on the effects of different flavored chewing gums and its effect on whole mouth SFR and pH in healthy individuals.

The aim of this study, therefore, was to assess the effect of five different flavors of sucrose-free chewing gum on the SFR and pH in healthy dental students in Isfahan, Iran.

## 2. Subjects and Methods

### 2.1. Participants

Following institutional ethical committee approval and informed participants consent, 15 dental student volunteers, in good general and oral health, (7 men and 8 women) mean aged 20.3 years, who fulfilled inclusion criteria, were approached to participate in this study. Exclusion criteria were smokers and have significant oral, dental, or systematic disease, taking any medication likely to interfere with salvation, wearing any intra-oral appliances, and having allergy to gum ingredients.

### 2.2. Chewing Gum

The five flavored chewing gums were used. The chewing gums tested were sucrose-free coating and contained 2% flavor compounds, 58% sweetener (xylitol and sorbitol), and 40% gum base, commercially available, spearmint-, cinnamon-, watermelon-, strawberry-, and apple-flavored (Orbit, Wrigley, Poland, Sp. z o.o, Poznan) purchased from local store. The pellets of each gum flavored were similar in volume, sweetener, and mass. Each pellet weighed 1.4 grams.

### 2.3. Saliva Collection

The participants were asked not to eat, drink, or chew gum for at least one hour prior to the saliva collection time. In order to avoid possible confounding effects of circadian rhythms in SFR, saliva collections were performed at the same time of six consecutive days (9–11 am) [7]. Unstimulated and gum-stimulated whole mouth saliva was collected 24 times from each participant. In each session before chewing any gum, unstimulated whole mouth saliva was collected from each participant. After 5 minutes, while some volunteers still continued to collect only unstimulated saliva, the other participants were asked to start chewing one pellet of the five flavored gums, at their natural chewing frequency. The whole mouth saliva was collected at intervals of 0-1, 1–3, and 3–6 min in unstimulated and after the start of chewing a single pellet of flavored gum in separate containers. For each subject, the order in which the five flavored gums were used was randomized, so every participant, over the six days, chewed all five flavors and collected his/her whole mouth unstimulated saliva over the same time periods. Collection of whole saliva was carried out through a disposable tube. Saliva was collected in the mouth and voided at regular intervals. This method tending to produce higher flow rates than the alternative method of continuous drainage from the open mouth. Saliva was allowed to dribble into a funnel and was collected in a graduated, disposable centrifuge tube. The tube was weighted before and after saliva collection. The amount of saliva was calculated as the difference between the two weights with two digits (1 g = 1 mL) and flow rate was calculated (mL/min). During these collection periods, the participants were instructed not to swallow any of their saliva; during noncollection periods they were allowed to swallow their saliva.

The interval between the different gum flavor experiments was 24 h to allow salivary flow rates and pH to return to basal levels.

The pH of the sampled saliva was also measured in unstimulated and before and after chewing gum. The pH was measured immediately after saliva collection in order to minimize any time-based pH changes, using a calibrated pH meter (Corning-450, Corning, NY, USA). The electrode was placed in the sample and the pH recorded to two decimal places.

### 2.4. Statistical Analysis

 The results for the groups that received unstimulated or gum-stimulated SFR and pH were compared with one-way ANOVA and analysis of variance with repeated measures over time; the results at baseline and after 6 minutes within each group were compared with paired Student's *t*-tests. The results are expressed as the mean (SD), and *P* < 0.05 was considered statistically significant. Analyses were initially stratified by gender, but as the findings were similar, the results are presented for both gender combined to increase statistical power. All statistical tests were two-sided. Analyses were done using SPSS for Windows (SPSS Inc., Chicago, IL, USA).

## 3. Results

**Figure 1:**
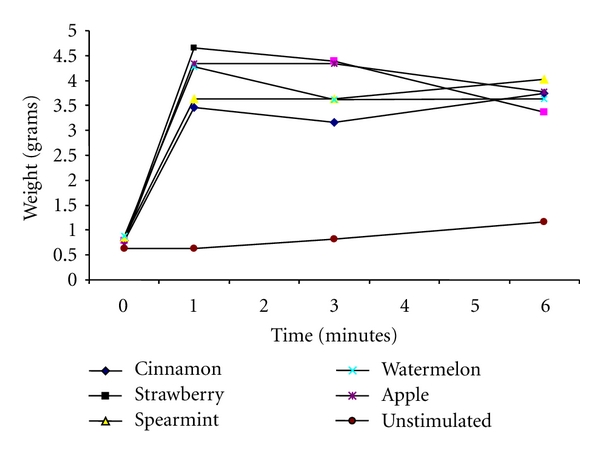
The changes in mean salivary flow rate (SFR) over 6 min after the start of one pellet of cinnamon-, strawberry-, spearmint-, watermelon-, or apple-flavored chewing gum compared with unstimulated SFR. The mean (SD) of SFR before and 0-1, 1–3 and 3–6 min after the start of different flavored chewing gums is shown.

Fifteen individuals who met the entry criteria were enrolled in the study. Participant's compliance with gum use was good. All 15 participants who completed trial were available for follow-up at 6 days. The mean SFR and salivary pH obtained on six different days did not show great variation.  Figure 1 and Table 1 shows the estimated marginal mean changes in SFR. The results for all unstimulated and gum-stimulated samples are set at the mid points of the collection periods; the value for 0–6 min sample was put at the mid points of 0-1, 1–3, and 3–6 min period, in order to avoid considerable overlap of the error bars in the figure. For initial unstimulated saliva sample collected at the beginning of each of the six collection sessions, there were no significant differences in SFR. The overall analysis of repeated measures ANOVA for unstimulated saliva showed significance difference in SFR with time (*P* < 0.01). Of the participants with unstimulated saliva, the mean (SD) SFR increased from 0.63 (0.37) at baseline to 1.17 (0.55) at the end of study period (*P* < 0.01).

Changes in mean SFR and pH before and 6 min after receiving flavored chewing gums are shown in Table 2. The average SFR increased significantly in all 5 flavored gums. The average SFR after 6 min chewing gum was higher in spearmint-flavored gum and lower in strawberry-flavored gum (4.03 mL/min and 3.36 mL/min resp.; *P* = 0.091). The peak salivary flows occurred in the first minute after the start of chewing and were 4.66 mL/min for strawberry-flavored gum followed by apple- and watermelon-flavored gum (4.35 and 4.29 mL/min, resp.). The peak salivary flows for spearmint-flavored were reached in the 6th min after the start of chewing. The mean flow rates for all flavored stimulated gum were greater than unstimulated flow rates for all time points (*P* < 0.001). The overall analysis of repeated measures ANOVA for 5 flavored-stimulated gums revealed significance differences in the SFR with time (*P* < 0.001).

There were no significant differences between salivary pH before stimulation by the five flavor groups. For the session in which only unstimulated saliva samples were collected, the mean salivary pH was relatively constant and in range of 6.17–6.40. Although all five different flavored chewing gums increased salivary pH, these values were significant only in cinnamon and spearmint flavors. When participants received cinnamon-flavored gum, the mean (SD) pH increased from 6.20 (0.53) at baseline to 7.40 (0.34) at the end of study period (*P* < 0.001). When participants received spearmint-flavored gum, the mean (SD) pH increased from 6.40 (0.47) at baseline to 7.53 (0.40) at the end of study period (*P* < 0.001) (Table 2). The cinnamon- or spearmint-flavored gums had about one whole pH unit greater than the pH of fruit-flavored gums. With fruit-flavored gums, the pH values slightly increased with each fruit-flavored gum pellet, but this effect was not statistically significant.

## 4. Discussion

In this study, while the 5 different sucrose-free coating chewing gum flavors were almost equally effective in stimulating SFR during the first, 1–3-, and 3–6-minute intervals, the salivary pH was greater with cinnamon- and spearmint-flavored gum.

In the present study, the strawberry-flavor caused slightly higher stimulation of SFR at 1st min stimulation. Previous data are inconsistent regarding the effects of variant flavors [4, 6, 8, 9]. Jensen et al. [9] reported that a cinnamon-flavored gum elicited more saliva than one flavored by peppermint, whereas in other studies no difference or very little differences were reported [4, 6]. The mechanisms whereby strawberry-flavored exerts higher stimulation on SFR are not clear. However, nasal chemosensory afferents may play a role for the salivary reflexes [10]. These warrant further studies.

Although all different flavored chewing gum increased salivary pH, these values were significant only with cinnamon- and spearmint-flavored gums. The increase in salivary pH on stimulation is due to the increase in bicarbonate concentration which is proportional to flow rate [11]. Consistent with previous studies [3, 4, 7, 8] which had evaluated mint- or cinnamon-flavored gums, we found that fruit-flavored gums lesser than cinnamon- and spearmint-flavored gum affect salivary pH. Fruit-flavored but not spearmint- and cinnamon-flavored gums contained citric and maleic acids, which can be responsible for less pH increase after chewing these fruity gums. On the other hand, presence of these two acids in fruity gums can lead to more salivary secretion after chewing these gums, compared with cinnamon- and spearmint-flavored gums.

Several investigators suggested the clinical use of sugar-free chewing gums for the relief of patients with xerostomia/hyposalivation [12–15]. Although all chewing gums investigated in our study stimulated the SFR significantly, the strawberry-flavored gum showed the highest SFR in the 1st and 3rd minute; apple- and watermelon-flavored gum followed it, respectively. Moreover at the end of 6 min after chewing strawberry-flavored gums, the mean SFR was yet 3 times greater than unstimulatory flow rate. So it can be suggested that in patients with hyposalivation, fruit-flavored gums can be advised to use, because of their more irritation of salivary secretion. On the other hand, in patients who are more susceptible to pH fall and dental caries, the use of spearmint- and cinnamon-flavored gums, which can raise the salivary pH significantly, is advisable.

This study could draw criticism because of the short follow-up period of 6 min. This may be short to appreciate the long-term impact of different flavored gum. Other limitations include the use of a relatively small sample of patients. Our study was limited by possible selection bias by restricting the study to dental students. The study tested only one brand of gum and therefore may not be representative of different brands of gums and could limit the generalizability of our findings. The efficacy should therefore be tested in a larger sample with a longer follow-up period and different brands of gums. The present results clearly need to be replicated and extended across multiple centers and investigators.

In conclusion, this comparative study of five different flavored gums provide further evidence that gum flavored can affect the SFR and pH and special flavors can be advised for different individuals according to their oral conditions.

## Figures and Tables

**Table 1:** The changes in mean salivary flow rate (SFR) over 6 min after the start of one pellet of cinnamon-, strawberry-, spearmint-, watermelon, or apple-flavored chewing gum compared with unstimulated SFR. The mean (SD) of SFR before and 0-1, 1–3 and 3–6 min after the start of different flavored chewing gums is shown (also see Figure 1).

Gum-flavored	Mean (SD) Salivary flow rate				*P *value
	Unstimulated	0-1 minute	1–3 minute	3–6 minute	
Cinnamon	0.79 (0.33)	3.46 (1.27)	3.17 (1.11)	3.76 (1.51)	0.001
Spearmint	0.85 (0.35)	3.63 (1.35)	3.63 (1.11)	4.03 (1.00)	0.001
Strawberry	0.78 (0.46)	4.66 (1.85)	4.38 (1.32)	3.36 (1.10)	0.001
Watermelon	0.88 (0.39)	4.29 (1.64)	3.62 (1.43)	3.63 (0.81)	0.001
Apple	0.71 (0.33)	4.35 (1.52)	4.34 (1.01)	3.78 (1.01)	0.001
Unstimulated	0.63 (0.37)	0.63 (0.37)	0.81 (0.35)	1.17 (0.55)	0.010

**Table 2:** Salivary flow rate and pH differences before and 6 min after the start of different flavored chewing gums.

Group	Before mean (SD)	After 6-minute mean (SD)	Differences (95% CI)	*P* value
	Salivary pH			
Cinnamon	6.20 (0.53)	7.40 (0.34)	1.20 (0.89, 1.51)	0.001
Spearmint	6.40 (0.47)	7.53 (0.40)	1.13 (0.94, 1.33)	0.001
Strawberry	6.17 (0.67)	6.30 (1.15)	0.13 (−0.63, 0.90)	0.710
Watermelon	6.17 (0.56)	6.50 (1.11)	0.33 (−0.36, 1.02)	0.319
Apple	6.23 (0.70)	6.27 (1.13)	0.04 (−0.47, 0.54)	0.890

	Salivary flow rate			
Cinnamon	0.79 (0.33)	3.76 (1.51)	3.01 (2.17, 3.74)	0.001
Spearmint	0.85 (0.35)	4.03 (1.00)	3.18 (2.65, 3.70)	0.001
Strawberry	0.78 (0.46)	3.36 (1.10)	2.58 (1.95, 3.21)	0.001
Watermelon	0.88 (0.39)	3.63 (0.81)	2.75 (2.24, 3.26)	0.001
Apple	0.71 (0.33)	3.78 (1.01)	3.07 (2.61, 3.53)	0.001
